# A systematic review of serum autoantibodies as biomarkers for pancreatic cancer detection

**DOI:** 10.18632/oncotarget.7098

**Published:** 2016-01-31

**Authors:** Karin Dumstrei, Hongda Chen, Hermann Brenner

**Affiliations:** ^1^ Division of Clinical Epidemiology and Aging Research, German Cancer Research Center (DKFZ), D-69120 Heidelberg, Germany; ^2^ European Molecular Biology Organization (EMBO), D-69117 Heidelberg, Germany; ^3^ Division of Preventive Oncology, German Cancer Research Center (DKFZ) and National Center for Tumor Diseases (NCT), D-69120 Heidelberg, Germany; ^4^ German Cancer Consortium (DKTK), German Cancer Research Center (DKFZ), D-69120 Heidelberg, Germany

**Keywords:** autoantibodies, biomarkers, pancreatic cancer, early detection, systematic review

## Abstract

Pancreatic cancer is a leading cause of cancer-related deaths in the western world. Patients with pancreatic cancer have poor prognosis, partly due to difficulties in detecting it at early stages. While different markers have been associated with pancreatic cancer, many of them show suboptimal sensitivity and specificity. Serum autoantibodies against tumor-associated antigens have recently emerged as early stage biomarkers for different types of cancers. Given the urgent need for early and reliable biomarkers for pancreatic cancer, we undertook a systematic review of the published literature to identify primary articles that evaluated serum autoantibodies in pancreatic cancer detection by searching PubMed and ISI Web of Knowledge. Two reviewers extracted data on study characteristics and results independently. Overall, 31 studies evaluating 124 individual serum autoantibodies in pancreatic cancer detection met the inclusion criteria. In general, single autoantibody markers showed relatively low sensitivities at high specificity. A combination of markers, either multiple serum autoantibodies or serum autoantibodies combined with tumor-associated markers, led to a better diagnostic performance. However, most of the analyzed autoantibodies have only been reported in single studies and therefore need to be independently validated. We conclude that serum autoantibodies might present an option as biomarkers for early detection of pancreatic cancer, but more work is needed to identify and validate autoantibody signatures that are associated with early stage pancreatic cancer.

## INTRODUCTION

Pancreatic cancer is one of the most common causes of cancer related deaths and represents a serious health problem. In the US, pancreatic cancer is the 4th leading cause of cancer death [1]. The vast majority of cases are seen in patients above the age of 55 with the median age of onset being 71 [2]. What makes the outlook for pancreatic cancer particularly troubling is the poor prognosis. The five-year relative survival rates are commonly below 10% and the incidence rate matches closely to the mortality rate. In the US, 46420 new cases were expected in 2014 while 39590 deaths were anticipated. One of the reasons for the poor prognosis is that most patients have locally advanced or metastatic cancer at time of diagnosis. 53% of patients are diagnosed at late stages with a 5-year survival rate of 2%, but even for the 9% percent of patients that are diagnosed with local cancer the 5-year relative survival rate is only 24% [1].

Most cases of pancreatic cancer are sporadic with the major risk factors being aging, smoking, diabetes, chronic pancreatitis and obesity [2, 3]. Inherited genetic factors are thought to contribute to 5–10% of pancreatic cancers [3–5]. Mutations in a number of different genes like, the familial breast cancer associated gene BRAC2 and its binding partner PALB2 lead to increased risk of developing pancreatic cancer. Some studies have also linked BRAC1 to pancreatic cancer, but the evidence is less strong as compared to BRAC2. Mutations in the cell cycle regulator *p16*/CDKN2A are also associated with familiar pancreatic cancer as are mutations in the serine/threonine kinase STK11/LKB1 [3–5].

Given the relative low incidence rate of pancreatic cancer, screening of the general population is in general not recommended. However, experts do agree on the benefit of screening patients that are at high risk for developing pancreatic cancer [6]. As there are currently no reliable biomarkers or screening tools available for detecting early stages of pancreatic cancer there is, however, no consensus for the most effective screening protocol

There have been many tumor-associated markers described for pancreatic cancer. Some of the better-characterized ones are CA19–9, CA-50 and CEA (reviewed in [7]). However, these markers tend to show suboptimal sensitivity and specificity [7]. CA19-9, for example, is also overexpressed in other types of gastrointestinal cancers and in inflammatory conditions such as pancreatitis and is thus not specific for pancreatic cancer [7]. There is therefore an urgent need to find better biomarkers and more accurate diagnostic tools.

The immune system also reacts to developing tumors and generates autoantibodies against tumor-associated antigens (TAA). This has led to a search for serological autoantibodies and their respective antigens in different types of cancers [8, 9]. While the mechanism of autoantibody production is not fully clear, cancer patients do produce autoantibodies to proteins that are either mutated, misfolded, overexpressed or to proteins that show altered post-translational modifications like glycosylation. Recent work supports that serum autoantibodies may be suitable biomarkers that can be used either alone or in combination with tumor associated markers or other autoantibodies for detection of cancers [8, 9]. The hope is that one can come up with defined autoantibody signatures for different types of cancers and tumor stages that can be used to detect cancers at early stages.

A number of studies have evaluated serological autoantibodies in pancreatic cancer. However, so far no comprehensive review of these studies has been done. We provide here a systematic review of the published literature to identify articles that have looked at serum autoantibodies in pancreatic cancer. We report the key aspects of the study design and population characteristics, the sensitivity and specificity of the investigated autoantibodies and marker combinations performed to provide a review of where the field stands at this stage.

## RESULTS

**Figure 1:**
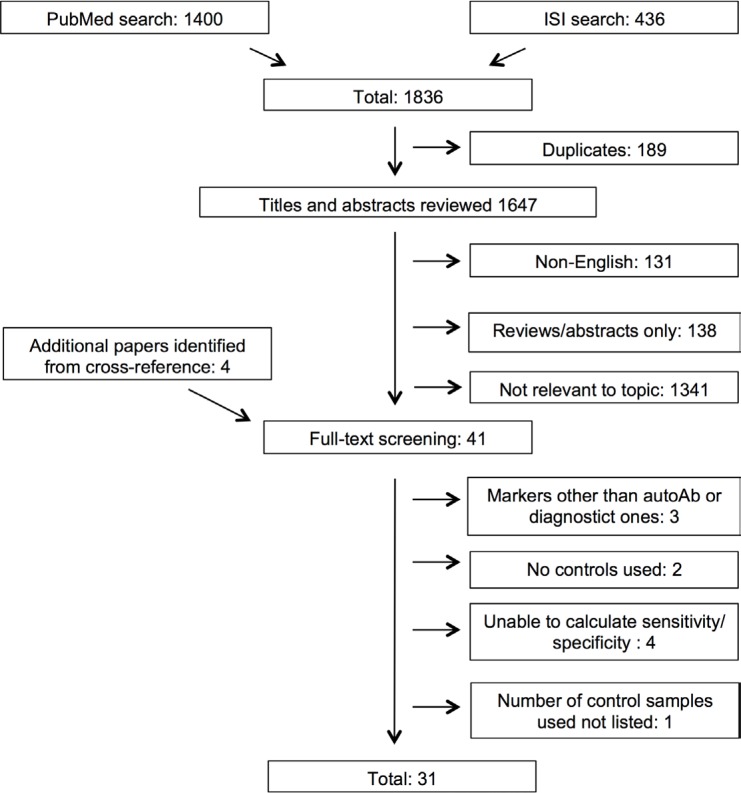
Overview of the literature search process (until 27th of April 2015)

The literature search process is shown in Figure 1. Overall, we identified 1836 articles using PubMed and Web of Science searches. Of these 189 were duplicates, 131 non-English and 138 were reviews/abstracts. Based upon title/abstract reading 1341 articles were not relevant to the topic leading to the full text screening of 41 articles. Ten of these were excluded due to the following reasons (see also Supplementary File S1): three studies evaluated markers other than autoantibodies or diagnostic ones [10–12], two studies lacked cancer free controls [13, 14], we were unable to calculate sensitivity and specificity in four studies [15–18] and one study did not provide the number of included controls [19] In the end, with four articles additionally identified through cross-referencing [20–23], 31 articles were included in our review [20–50].

### Study characteristics

The key study characteristics are provided in Supplementary Table S2. This table shows for each study the number of cases and controls, the age range and average age, male/female ratio, the status of the controls as well as the detection method used to evaluate serum autoantibodies. 10 studies [20, 23–25, 28, 29, 42, 44, 47, 48] provided complete information on all these variables. The median numbers of pancreatic cases and corresponding controls included were 47 (range: 8–300) and 43 (range: 5–436), respectively.

Healthy controls were used in 25 studies, while in the other studies a mixture of patients with non-cancer pancreatic diseases (e.g., chronic pancreatitis) were used. One study used a random random-digit dial method to select controls and didn't specify the status of the controls in terms of non-cancer pancreatic diseases or other types of cancers [24]. Tumor stage information was provided in two studies [20, 35]. The age range and average age were reported in 10 studies, while in 14 studies the average age was provided. In studies where the age information was provided, most of them showed a fairly similar age distribution among cases and controls.

Different techniques were used to detect serum autoantibodies with the most common one being ELISA (17 studies). Western blot analysis and different proteomic approaches were also used in some studies. Within the last five years the use of proteomic analysis has become a more frequent choice for this type of analysis. In most studies, recombinant full-length proteins were used as antigen for autoantibodies. However in some studies, peptides [39, 45, 46] and glycosylated proteins [25] also served as antigens.

### Diagnostic performance of autoantibodies

**Figure 2:**
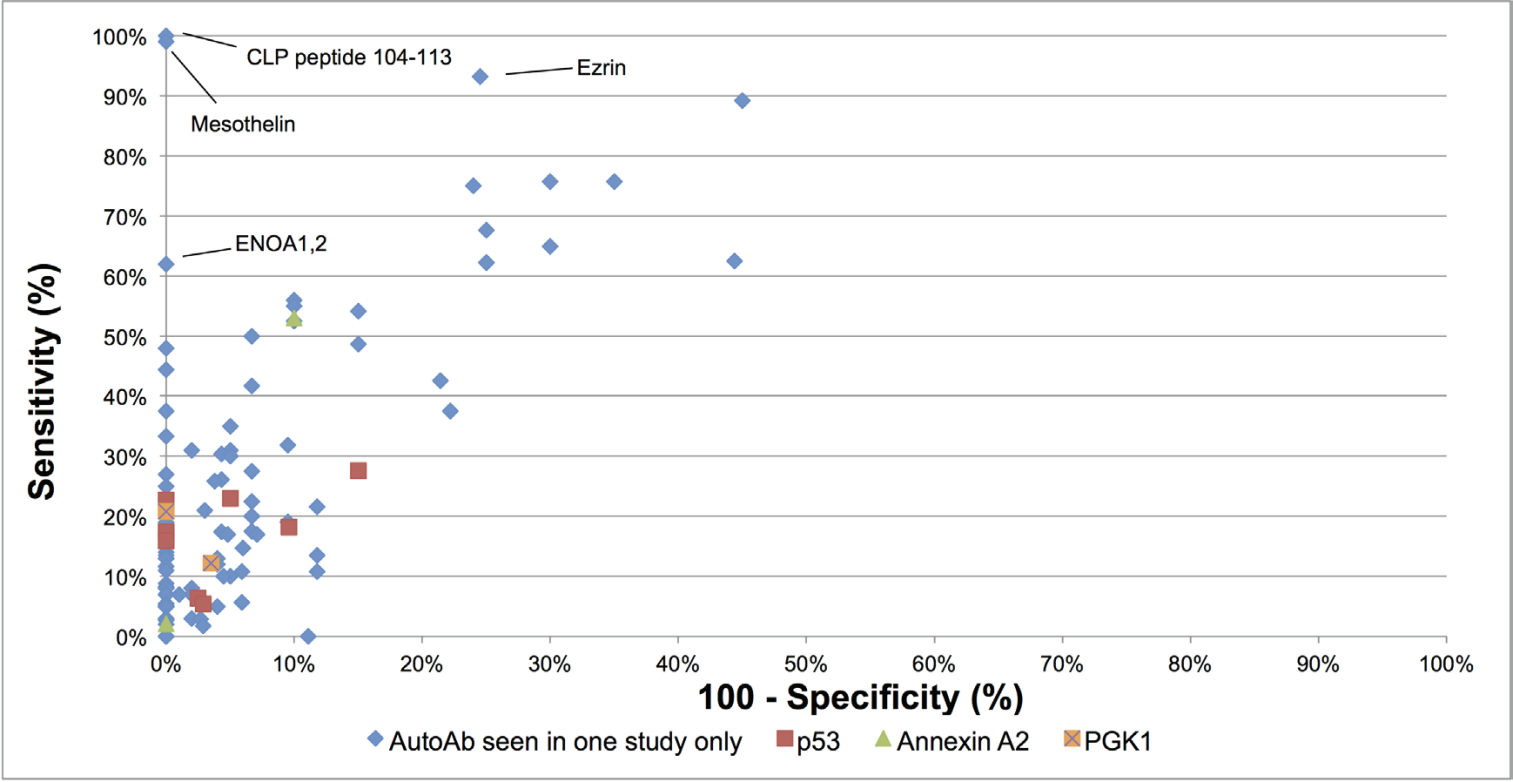
Graphical representation of sensitivity versus specificity of all analyzed autoantibodies Sensitivity is plotted on the *y*-axis while on the *x*-axis the false positive rate is presented (100 - Specificity). Autoantibodies evaluated in one study only are labeled in blue. Autoantibodies evaluated in more than one study are labeled in red (p53), green (Annexin A2) and orange (PGK1). Autoantibodies that showed high sensitivity and specificity are labeled directly on the graph. Abbreviations: CLP: Coactosin-like protein; ENOA: Alpha-enolase; PGK1: Phosphoglyceratekinase 1.

Overall, 124 individual autoantibodies were evaluated in the 31 included studies. The diagnostic performance of these autoantibodies, ordered by reported sensitivity, is listed in Table 1. The diagnostic performance of the autoantibodies varied greatly in terms of sensitivity and specificity. The sensitivity ranged from 0% to 100% with a median of 14% (average is 22%). In general, the majority of markers showed a relative low sensitivity. 105 of the examined autoantibodies (85%) showed less than 50% sensitivity. The specificity ranged from 55% to 100% with a median of 100% (average 95%) and 85% of autoantibodies showed specificity greater or equal to 90%. Four autoantibodies showed high specificity (> 80%) along with high sensitivity (> 60%). These markers are anti-Coactosin-like protein (CLP) peptide 104–113 [39], anti-Mesothelin [32], anti-Ezrin [26] and anti-ENOA1,2 [48] – see also Figure 2. However, it is important to note that the diagnostic performance of these autoantibodies has not been validated in other independent studies and case numbers in some of the studies were very small. AUC values were reported for 15 autoantibodies, but no internal or external validations were applied to adjust for potential over-optimism. Figure 2 shows a graphical representation of the sensitivities and specificities for all examined autoantibodies. As can be seen from Figure 2 as well as from Table 1, autoantibodies that showed high sensitivity tended to show lower specificity. Conversely, markers with low sensitivity tended to have high specificity.

**Table 1:** Diagnostic performance of antibodies markers ordered by reported sensitivity

First author, Year [Ref]	Antigen	Cases (N)/Controls (N)	Sensitivity % (95% CI)	Specificity % (95% CI)	AUC	*p*-value*
Nakatsura, 2002 [39]	CLP peptide 104–113 IgG	8/9	100 (63–100)	100 (66–100)	–	–
Johnston, 2009 [32]	Mesothelin	74/5	99 (93–100)	100 (48–100)	–	*p* < 0.05
Capello, 2013 [26]	Ezrin	69/94	93 (83–98)	76 (66–84)	0.9	*p* < 0.0001
Nagayoshi, 2014 [38]	TNP1	37/20	89 (75–97)	55 (32–77)	0.732	–
Nagayoshi, 2014 [38]	CIB1	37/20	76 (59–88)	70 (46–88)	0.753	–
Nagayoshi, 2014 [38]	RIT2	37/20	76 (59–88)	65 (41–85)	0.704	–
Falco, 2013 [27]	Bag3	52/44	75 (61–86)	76 (60–87)	0.77	*p* = 0.00001
Nagayoshi, 2014 [38]	GABARAPL2	37/20	68 (50–82)	75 (51–91)	0.674	–
Nagayoshi, 2014 [38]	KIAA0409	37/20	65 (47–80)	70 (46–88)	0.72	–
Nakatsura, 2002 [39]	CLP peptide 15–24 IgE	8/9	63 (24–91)	56 (21–86)	–	–
Nagayoshi, 2014 [38]	DTYMK	37/20	62 (45–78)	75 (51–91)	0.691	–
Nagayoshi, 2014 [38]	STK33	37/20	62 (45–78)	75 (51–91)	0.668	–
Tomaino, 2011 [48]	ENOA1,2	61/63	62 (49–74)	100 (94–100)	–	*p* = 0.0001
Capello, 2013 [26]	Ezrin	120/40	56 (46–65)	90 (76–97)	–	*p* < 0.0001
Tanaka, 2007 [45]	PSCA peptide 2–11	40/60	55 (38–71)	90 (79–96)	–	*p* = 0.0001
Nagayoshi, 2014 [38]	PCNA	37/20	54 (37–71)	85 (62–97)	0.669	–
Capello, 2013 [26]	Annexin A2	120/40	53 (44–62)	90 (76–97)	–	*p* < 0.0001
Tanaka, 2007 [45]	PSCA peptide 86–95	40/60	53 (36–68)	90 (79–96)	–	*p* = 0.0001
Kamei, 1992 [33]	Histone H2B	8/45	50 (16–84)	93 (82–99)	–	–
Nagayoshi, 2014 [38]	EIF3S4	37/20	49 (32–66)	85 (62–97)	0.67	–
Syrigos, 1996 [44]	Insulin	36/21	48 (30–65)	100 (84–100)	–	–
Hong, 2004 [31]	Calreticulin isoform 2	36/15	44 (28–62)	100 (78–100)	–	–
Tanaka, 2006 [46]	SART-109 peptide	47/42	43 (28–58)	79 (63–90)	–	*p* < 0.05
Hong, 2004 [31]	Calreticulin isoform 1	36/15	42 (26–60)	93 (68–100)	–	–
Capello, 2013 [26]	Ezrin	16/32	38 (15–65)	100 (89–100)	–	*p* = 0.0002
Nakatsura, 2002 [39]	CLP peptide 104–113 IgE	8/9	37 (9–76)	78 (40–97)	–	–
Capello, 2013 [26]	hnRNPL	120/40	35 (27–44)	95 (83–99)	–	*p* < 0.001
Xia, 2005 [49]	DDX48	60/60	33 (22–47)	100 (94–100)	–	*p* < 0.01
Tanaka, 2006 [46]	EGFR-479 peptide	47/42	32 (19–47)	91 (77–97)	–	*p* < 0.05
Capello, 2013 [26]	Vinculin	120/40	31 (23–40)	95 (83–99)	–	*p* = 0005
Pekarikova, 2010 [23]	Calreticulin IgG	55/56	31 (19–45)	98 (90–100)	–	–
Li, 2012 [35]	p16	23/23	30 (13–53)	96 (78–100)	–	*p* < 0.05
Tanaka, 2007 [45]	PSCA peptide 109–118	40/60	30 (17–47)	95 (86–99)	–	–
Laurent-Puig, 1995 [34]	p53	29/33	28 (13–47)	85 (68–95)	–	–
Tanaka, 2007 [45]	PSCA peptide 108–117	40/60	28 (15–44)	93 (84–98)	–	*p* = 0.0431
Tomaino, 2007 [47]	TAGL or COF1	70/40	27 (17–39)	100 (91–100)	–	*p* = 0.002
Li, 2012 [35]	IMP1	23/23	26 (10–48)	96 (78–100)	–	–
Patwa, 2009 [42]	Histone H4	54/94	54 (15–40)	96 (89–99)	–	–
Pekarikova, 2010 [23]	Calreticulin IgA	55/56	25 (15–39)	100 (94–100)	–	–
Ohshio, 2002 [40]	p53	82/21	23 (15–34)	95 (76–100)	–	–
Tomaino, 2007 [47]	TPIS	70/40	23 (14–34)	100 (91–100)	–	*p* = 0.004
Muller, 2006 [37]	p53	22/436	23 (8–45)	100 (99–100)	–	–
Tanaka, 2007 [45]	PSCA peptide 18–27	40/60	23 (11–38)	93 (84–98)	–	*p* = 0.0105
Li, 2012 [35]	p62	23/23	22 (7–44)	100 (85–100)	–	–
Li, 2012 [35]	Koc	23/23	22 (7–44)	100 (85–100)	–	–
Okada, 2005 [41]	Kinectin1	37/34	22 (10–38)	88 (73–97)	–	–
Capello, 2013 [26]	PDC6I	120/40	21 (14–29)	97 (87–100)	–	*p* = 0.0033
Tomaino, 2007 [47]	K1C10	70/40	21 (13–33)	100 (91–100)	–	*p* = 0.005
Li, 2010 [21]	PGK1	48/40	21 (10–35)	100 (91–100)	–	–
Tanaka, 2007 [45]	PSCA peptide 51–60	40/60	20 (9–36)	93 (84–98)	–	*p* = 0.0155
Tanaka, 2007 [45]	PSCA peptide 27–37	40/60	20 (9–36)	93 (84–98)	–	–
Tomaino, 2007 [47]	AL1A1	70/40	20 (11–31)	100 (91–100)	–	*p* = 0.006
Tanaka, 2006 [46]	Pap-112 peptide	47/42	19 (9–33)	91 (77–97)	–	*p* > 0.05
Capello, 2013 [26]	Annexin A1	120/40	19 (13–27)	100 (91–100)	–	*p* = 0.0012
Raedle, 1996 [43]	p53	33/52	18 (7–35)	90 (79–97)	–	–
Tanaka, 2007 [45]	PSCA peptide 44–52	40/60	18 (7–33)	93 (84–98)	–	*p* = 0.0398
Li, 2012 [35]	p53	23/23	17 (5–39)	100 (85–100)	–	–
Li, 2012 [35]	Survivin	23/23	17 (5–39)	96 (78–100)	–	–
Tanaka, 2006 [46]	EGFR-54 peptide	47/42	17 (8–31)	95 (84–99)	–	*p* > 0.05
Tanaka, 2006 [46]	CEA-425 peptide	47/42	17 (8–31)	93 (81–99)	–	*p* > 0.05
Li, 2010 [21]	MDH1	48/40	17 (8–31)	100 (91–100)	–	–
Gansange, 1996 [20]	p53	145/60	16 (10–23)	100 (94–100)	–	–
Heller, 2010 [30]	MIA	34/20	15 (5–31)	94 (75–100)	–	–
Tomaino, 2007 [47]	TPIS	70/40	14 (7–25)	100 (91–100)	–	*p* = 0.004
Okada, 2005 [41]	hMSH2	37/34	14 (5–29)	100 (90–100)	–	–
Okada, 2005 [41]	IMAGE:3480396 3′	37/34	14 (5–29)	88 (73–97)	–	–
Gnjatic, 2010 [29]	NR2E3	60/53	13 (6–25)	96 (87–100)	–	–
Tomaino, 2007 [47]	G6PD	70/40	13 (6–23)	100 (91–100)	–	*p* = 0.03
Tomaino, 2007 [47]	IDHC	70/40	13 (6–23)	100 (91–100)	–	*p* = 0.03
Patwa, 2009 [42]	PGK1	49/43	12 (5–25)	97 (88–100)	–	–
Gnjatic, 2010 [29]	ROR2	60/53	12 (5–23)	96 (87–100)	–	–
Heller, 2010 [30]	PNLIPRP2	34/20	12 (3–28)	100 (83–100)	–	–
Tomaino, 2007 [47]	EFTU	70/40	11 (5–21)	100 (91–100)	–	*p* = 0.04
Okada, 2005 [41]	KIAA0580	37/34	11 (3–25)	94 (80–99)	–	–
Okada, 2005 [41]	RUNX2	37/34	1 (3–25)	88 (73–97)	–	–
Tanaka, 2007 [45]	PSCA peptide 3–11	40/60	10 (3–24)	95 (86–99)	–	*p* = 0.0000
Tanaka, 2007 [45]	PSCA peptide 3–12	40/60	10 (3–24)	96 (88–100)	–	*p* = 0.0000
Heller, 2010 [30]	IFITM3	34/20	9 (2–24)	100 (83–100)	–	–
Li, 2010 [21]	ARFIP2	48/40	8 (2–20)	100 (91–100)	–	–
Okada, 2005 [41]	hPMS1	37/34	8 (2–22)	100 (90–100)	–	–
Okada, 2005 [41]	HRY	37/34	8 (2–22)	100 (90–100)	–	–
Gnjatic, 2010 [29]	MAPK9	60/53	8 (3–18)	100 (93–100)	–	–
Gnjatic, 2010 [29]	C6orf141	60/53	8 (3–18)	100 (93–100)	–	–
Gnjatic, 2010 [29]	MAPK9	60/53	8 (3–18)	100 (93–100)	–	–
Gnjatic, 2010 [29]	GAS2	60/53	8 (3–18)	98 (90–100)	–	–
Gnjatic, 2010 [29]	KIAA1618	60/53	8 (3–18)	98 (90–100)	–	–
Gnjatic, 2010 [29]	PTPRA	60/53	7 (2–16)	100 (93–100)	–	–
Gnjatic, 2010 [29]	LRRC49	60/53	7 (2–16)	100 (93–100)	–	–
Gnjatic, 2010 [29]	ULK4	60/53	7 (2–16)	100 (93–100)	–	–
Gnjatic, 2010 [29]	TMOD1	60/53	7 (2–16)	98 (90–100)	–	–
Gnjatic, 2010 [29]	C8orf34	60/53	7 (2–16)	100 (93–100)	–	–
Maacke, 2002 [22]	Rad51	57/86	7 (2–17)	100 (96–100)	–	–
Zhu, 2015 [50]	Brca1	41/135	7 (2–20)	99 (96–100)	–	*p* < 0.05
Marxsen, 1994 [36]	p53	78/82	6 (2–14)	98 (91–100)	–	–
Fyssas, 1997 [28]	thyroglobulin	33/40	6 (1–20)	98 (87–100)	–	–
Burfold, 2013 [25]	Muc1core3 glycopeptide	35/247	6 (1–19)	94 (90–97)	–	*p* > 0.5
Okada, 2005 [41]	SOX13	37/34	5 (1–19)	100 (90–100)	–	–
Okada, 2005 [41]	MRPL12	37/34	5 (1–19)	100 (90–100)	–	–
Okada, 2005 [41]	HMT1	37/34	5 (1–19)	100 (90–100)	–	–
Okada, 2005 [41]	Tim44	37/34	5 (1–19)	97 (85–100)	–	–
Okada, 2005 [41]	p53	37/34	5 (1–19)	97 (85–100)	–	–
Gnjatic, 2010 [29]	FAM13A1	60/53	5 (1–14)	100 (93–100)	–	–
Gnjatic, 2010 [29]	C17orf46	60/53	5 (1–14)	100 (93–100)	–	–
Gnjatic, 2010 [29]	HERPUD1	60/53	5 (1–14)	100 (93–100)	–	–
Gnjatic, 2010 [29]	AFG3L1	60/53	5 (1–14)	100 (93–100)	–	–
Gnjatic, 2010 [29]	C4orf16	60/53	5 (1–14)	100 (93–100)	–	–
Gnjatic, 2010 [29]	CD79B	60/53	5 (1–14)	100 (93–100)	–	–
Gnjatic, 2010 [29]	CRSP8	60/53	5 (1–14)	100 (93–100)	–	–
Gnjatic, 2010 [29]	DNAJB1	60/53	5 (1–14)	100 (93–100)	–	–
Gnjatic, 2010 [29]	NY-SAR–48	60/53	5 (1–14)	100 (93–100)	–	–
Gnjatic, 2010 [29]	PPARG	60/53	5 (1–14)	100 (93–100)	–	–
Gnjatic, 2010 [29]	SHOC2	60/53	5 (1–14)	96 (87–100)	–	–
Gnjatic, 2010 [29]	SMOX	60/53	5 (1–14)	100 (93–100)	–	–
Gnjatic, 2010 [29]	TMSB10	60/53	5 (1–14)	100 (93–100)	–	–
Gnjatic, 2010 [29]	ZNF695	60/53	5 (1–14)	100 (93–100)	–	–
Gnjatic, 2010 [29]	CRYBB2	60/53	3 (0–12)	98 (90–100)	–	–
Gnjatic, 2010 [29]	ELAC1	60/53	3 (0–12)	100 (93–100)	–	–
Gnjatic, 2010 [29]	HCFC1R1	60/53	3 (0–12)	100 (93–100)	–	–
Burfold, 2013 [25]	Muc1STn glycopeptide	35/247	3 (0–15)	97 (94–99)	–	*p* > 0.5
Okada, 2005 [41]	HAX1	37/34	3 (0–14)	100 (90–100)	–	–
Okada, 2005 [41]	ZNF207	37/34	3 (0–14)	100 (90–100)	–	–
Okada, 2005 [41]	RP-43L2	37/34	3 (0–14)	100 (90–100)	–	–
Li, 2010 [21]	Annexin A2	48/40	2 (0–11)	100 (91–100)	–	–
Pekarikova, 2010 [23]	tTG	55/56	2 (2–10)	100 (94–100)	–	–
Johnston, 2009 [32]	Mesothelin IgG	56/35	0 (0–10)	97 (85–100)	> 0.05	–
Li, 2010 [21]	HNRPA2	48/40	0 (0–7)	100 (91–100)	–	–
Nakatsura, 2002 [39]	CLP peptide 57–65 IgG	8/9	0 (0–37)	100 (66–100)	–	–
Nakatsura, 2002 [39]	CLP peptide 57–65 IgE	8/9	0 (0–37)	89 (52–100)	–	–
Zhu, 2015 [50]	Parp1	41/135	0 (0–9)	99 (96–100)	–	–
Zhu, 2015 [50]	Brca2	41/135	0 (0–9)	99 (96–100)	–	–
Bracci, 2012 [24]	NR2E3	300/300	–	–	0.56	–
Bracci, 2012 [24]	MAPK9	300/300	–	–	0.59	–
Bracci, 2012 [24]	CTDSP1	300/300	–	–	0.62	–

Abbreviations: AFG3L1: AFG3 ATPase family gene 3-like 1; AL1A1: Retinal dehydrogenase 1; ARFIP2: ADP-ribosylation factor interacting protein 2; CEA: Carcinoembryonic antigen; CI: Confidence interval; CIB1: Calcium and integrin binding 1; COF1: Cofilin-1; CLP: Coactosin-like protein; CRYBB2: Crystallin beta B2; CTDSP1: Carboxy-terminal domain RNA polymerase II polypeptide A small phosphatase 1; DDX48: Dead-box protein48; DNAJB1: DnaJ (Hsp40) homolog subfamily B member 1; EGFR: epidermal growth factor receptor; EFTU: Elongation Factor Tu; ELAC1: ElaC homolog 1, ENOA: Alpha-enolase; HRY: hairy Drosophila-homolog; IDHC: Isocitrate dehydrogenase; FAM13A1: Family with sequence similarity 13 member A; GAS2: Growth arrest-specific 2; G6PD: Glucose-6-phosphate 1-dehydrogenase; HAX1: HS1 binding protein; HCFC1R1: Host cell factor C1 regulator 1; HERPUD1: Homocysteine-inducible endoplasmic reticulum stress-inducible ubiquitin-like domain member 1; hPMS1: Homo sapiens postmeiotic segregation increased 1; hMSH2: Homo sapiens mutS homolog 2; HMT1: hnRNP methyltransferase; hnRNPL: Heterogeneous nuclear ribonucleoprotein L; HNRPA2: Heterogeneous nuclear ribonucleoprotein A2; IFITM3: Interferon-induced transmembrane protein 3; IMP1: IGF-II mRNA binding protein 1; K1C10: Keratin 10; Koc: KH-domain containing protein over expressed in cancer; LRRC49: Leucine rich repeat containing 49; MAPK: Mitogen-activated protein kinase; MDH1: Malate dehydrogenase; MIA: Melanoma-inhibitory activity; Muc1: Mucin 1; MRPL12: Mitochondrial ribosome protein L12; NR2E3: Nuclear receptor subfamily 2- group E- member 3; PARP1: poly (ADP-Ribose) Polymerase 1; PDC6I: Programmed Cell Death-6 Interacting protein; PGK1: Phosphoglyceratekinase 1; PNLIPRP2: Pancreas lipase-related protein 2; PPARG: Peroxisome proliferative activated receptor gamma; PSCA: Prostate stem cell antigen; PTPRA: Protein tyrosine phosphatase receptor type A; ROR2: Receptor tyrosine kinase-like orphan receptor 2; RPL29: 60S ribosomal protein L29, SHOC2: Soc-2 suppressor of clear homolog; SOX13: Sex determining region Y-box 13; TIM44: Translocase of inner mitochondrial membrane 44; TMSB10: Thymosin beta 10; tTG: Tissue transglutamase; SMOX: Spermine oxidase; TMOD1: Tropomodulin 1; TPIS: Triosephosphateisomerase 1; TAGL: Transgelin; ZNF207: Zinc finger protein 207; ZNF695: Zinc finger protein 695.

* *p*-value represents the statistical difference of positivity rate between cases and controls.

Three autoantibodies, anti-p53, anti-PGK1 and anti-Annexin A2, were examined in multiple studies (Table 2). Given the prominent role of p53 in multiple cancers it is perhaps not surprising that this was the most commonly assessed autoantibody. Autoantibodies against p53 were evaluated in 8 studies [20, 34–37, 40, 41, 43]. As can be seen from Table 2, the sensitivities reported for autoantibodies against p53 varied between studies (6–28%) while there was less variance in the specificity (range 85–90%). PGK1 [21, 42] and Annexin A2 [21, 26] were each evaluated in two studies and both show relative low sensitivity, but high specificity.

**Table 2:** Autoantibodies examined in multiple studies

Antigen	References	No. of Studies	Range across studies	
			Sensitivity (%)	Specificity (%)
p53	[20, 34–37, 40, 41, 43]	8	6−28	85−90
PGK1	[21, 42]	2	12−21	97−100
Annexin A2	[21, 26]	2	2−19	90−100

Abbreviations: PGK1 Phosphoglyceratekinase 1

Several studies also reported the sensitivity and specificity of a combination of different markers (Table 3). These studies looked at either multiple autoantibodies or autoantibodies combined with other tumor associated markers. The combination of markers generally led to enhanced sensitivity while maintaining relatively high specificity. For example the combination of anti-Ezrin and anti-ENOA1.2 with the tumor associated marker CA19.9 [26] lead to a sensitivity of 100%. Again, as observed with individual autoantibodies, autoantibodies against p53 were a common choice for the maker combinations. The two most commonly used markers were anti-p53 and CA19.9, which were included in 11 and 8 of the 31 possible multi-marker combinations, respectively. What is also evident from this analysis is the variability between different studies. Three studies looked at anti-p53 in combination with CA19.9 [37, 40, 43] and the reported sensitivity ranged from 26% to 73%, while the specificity varied from 64% to 100%.

**Table 3:** Diagnostic performance of marker combinations

First author, Year [Ref]	Marker Combination	Cases (N)/Controls (N)	Sensitivity % (95%CI)	Specificity % (95%CI)	AUC	*p*-value*
Capello, 2013 [26]	anti-Ezrin + anti-ENOA1.2 + CA19,9	45/48	100 (92–100)	92 (80–98)	0.96	–
Tomaino, 2011 [48]	anti-ENOA1,2 + CA19.9**	37/63	97 (86–100)	92 (82–97)	0.95	*p* = 0.0001
Tomaino, 2011 [48]	anti-ENOA1 + CA19.9***	61/63	95 (86–99)	94 (85–98)	0.94	*p* = 0.0001
Tanaka, 2006 [46]	anti-SART-109 + anti-EGFR-479 + anti-Pap-112 + anti-EGFR-54 + anti-CEA-425****	47/43	83 (69–92)	88 (75–96)	–	–
Tanaka, 2007 [45]	anti-PSCA peptide 2–11 + anti-PSCA peptide 86–95 + anti-PSCA peptide 109–118	40/60	80 (64–91)	82 (70–90)	–	–
Syrigos, 1996 [44]	anti-Pancreatic islet b-cells + anti-Insulin	36/21	73 (55–86)	100 (84–100)	–	*p* < 0.001
Raedle, 1996 [43]	anti-p53 + CA19.9 (Cutoff 37U/ml)	33/52	73 (54–87)	64 (50–76)	–	–
Li, 2012 [35]	anti-p53 + anti-p16 + anti-p62 + anti-Survivin + anti-Koc + anti-IMP1 +CA19.9	23/23	70 (47–87)	–	–	–
Tanaka, 2006 [46]	anti-SART-109 + anti-EGFR-479 + anti-Pap-112 + anti-EGFR-54 + anti-CEA-425 *****	47/42	64 (49–77)	71 (55–84)	–	–
Li, 2012 [35]	anti-p53 + anti-p16 + anti-p62 + anti-survivin + anti-Koc + anti-IMP1	23/23	61 (39–80)	87 (66–97)	–	*p* < 0.01
Hong, 2004 [31]	anti-Calreticulin isoform 1 + anti-Calreticulin isoform 2	36/15	58 (41–74)	93 (68–100)	–	–
Raedle, 1996 [43]	anti-p53 + CA19.9 (Cutoff 100U/ml)	33/52	58 (39–75)	87 (74–94)	–	–
Li, 2012 [35]	anti-p53 + anti-p16 + anti-p62 + anti-survivin + anti-Koc	23/23	52 (31–73)	91 (72–99)	–	–
Muller, 2006 [37]	anti-p53 + CA19–9	22/436	50 (28–72)	100 (99–100)	–	–
Li, 2012 [35]	anti-p53 + anti-p16 + anti-p62 + anti-survivin	23/23	48 (27–69)	95 (77–100)	–	–
Pekarikova, 2010 [23]	anti-Calreticulin IgA + anti-Calreticulin IgG	55/56	47 (34–61)	98 (90–100)	–	–
Li, 2010 [21]	anti-PGK1 + anti-MPH1 + anti-ARFIP2	48/41	46 (31–61)	100 (91–100)	–	–
Li, 2012 [35]	anti-p53 + anti-p16 + anti-p62	23/23	39 (20–61)	96 (78–100)	–	–
Li, 2010 [21]	anti-PGK1 + anti-MPH1	48/40	28 (24–53)	100 (91–100)	–	–
Li, 2012 [35]	anti-p53 + anti-p16	23/23	35 (16–57)	96 (78–100)	–	–
Patwa, 2009 [42]	anti-Histone H4 + anti-PGK1	−/−	33	94	–	–
Heller, 2010 [30]	anti-MIA + anti-PNLIPR2 + anti-IFITM2	34/20	32 (17–51)	94 (75–100)	–	*p* = 0.021
Syrigos, 1996 [44]	anti-Pancreatic islet b-cells and anti-Insulin	36/21	31 (16–48)	100 (84–100)	–	*p* < 0.001
Ohshio, 2002 [40]	anti-p53 and CA19.9	82/21	26 (17–36)	–	–	–
Fyssas, 1997 [28]	anti-Microsomes + anti-Thyroglobulin	33/40	25 (11–42)	95 (76–100)	–	–
Ohshio, 2002 [40]	anti-p53 and CEA	82/21	22 (14–32)	–	–	–
Fyssas, 1997 [28]	anti-Microsomes and anti-Thyroglobulin	33/40	3 (0–16)	98 (87–100)	–	–
Zhu, 2015 [50]	Parp1 and Brca1	41/135	0 (0–9)	100 (97–100)	–	–
Zhu, 2015 [50]	Parp1 and Brca2	41/136	0 (0–9)	100 (97–100)	–	–
Zhu, 2015 [50]	Brac1 and Brca2	41/137	0 (0–9)	100 (97–100)	–	–
Zhu, 2015 [50]	Parb1 and Brca1 and Brca2	41/138	0 (0–9)	100 (97–100)	–	–

+ denotes and/or

* *p*-value represents the difference of positivity rate between cases and controls.

** validation set

*** training set

**** Discriminatory analysis

***** Cumulative analysis

Abbreviations: ARFIP2: ADP-ribosylation factor interacting protein 2; CEA: Carcinoembryonic antigen; CI: Confidence Interval; EGFR: epidermal growth factor receptor; ENOA: Alpha-enolase; IFITM3: Interferon-induced transmembrane protein 3; IMP1: IGF-II mRNA binding protein 1; Koc: KH-domain containing protein over expressed in cancer; MIA: Melanoma-inhibitory activity PGK1: PARP1: poly (ADP-Ribose) Polymerase 1; Phosphoglyceratekinase 1; PNLIPRP2: Pancreas lipase-related protein 2; PSCA: Prostate stem cell antigen.

Two studies also reported sensitivity according to tumor stage [20, 35] (Supplementary Table S3) for 6 different autoantibodies. From this analysis, one can see that the diagnostic performance of the autoantibodies was mostly higher in more advanced tumor stages. The sensitivity for autoantibodies at tumor stages I and II was in the range from 3% to 13% with the exception of anti-p16 that showed a sensitivity of 33% at tumor stage I. More advanced stages (III and IV) showed higher sensitivity for all the investigated autoantibodies. One study also assessed the tumor stage specific sensitivity of a combination of 6 different autoantibodies [35]. As can be seen from Supplementary Table S3 this led to enhanced sensitivity at all stages from 33% at stage I to 86% at stage IV [35]. Both studies also evaluated the tumor stage specific sensitivity for autoantibodies against p53 and again here some variability was seen between the studies.

Previous work has also carried out systematic reviews on the diagnostic performance of serum autoantibodies in colorectal cancer [51] and gastric cancer [52]. Table 4 shows a comparison of the diagnostic performance of 9 different serum autoantibodies that have been analyzed in three gastrointestinal cancers. None of these markers seemed to be specific for one of the cancers only.

**Table 4:** Comparison of diagnostic performance of autoantibodies in detecting three different cancers

Antigen	Test characteristic	This Study	Chen et al. [51]	Werner et al. [52]
		Pancreatic cancer	Colorectal cancer	Gastric cancer
p53	Sensitivity	5%–28%	9–46%	8–32%
	Specificity	85%–90%	90–100%	95–100%
Histone H2B	Sensitivity	50%	30%	17%
	Specificity	93%	92%	93%
p16	Sensitivity	30%	11%	11%
	Specificity	96%	99%	99%
p62	Sensitivity	22%	9–23%	7–9%
	Specificity	100%	97–99%	98%
Survivin	Sensitivity	17%	4–56%	8–10%
	Specificity	96%	64–98%	98%
Koc	Sensitivity	22%	9–15%	16–19%
	Specificity	100%	99–100%	98%
CEA	Sensitivity	17%	9–64%	52%
	Specificity	93%	89–96%	89%
DDX48	Sensitivity	33%	10%	7%
	Specificity	100%	100%	100%
IMP1	Sensitivity	26%	13–22%	17%
	Specificity	96%	98–100%	98%

Comparison of diagnostic performance of autoantibodies found in pancreatic cancer (this study), in colorectal cancer [52] and in gastric cancer [53]. Abbreviations: CEA: Carcinoembryonic antigen; DDX48: Dead–box protein48; Koc: KH–domain containing protein over expressed in cancer; IMP1: IGF–II mRNA binding protein.

## DISCUSSION

In this systematic literature review, we identified 31 studies on serum autoantibodies for the detection of pancreatic cancer that fit our inclusion criteria. The identified studies evaluated the diagnostic performance of 124 different serum autoantibodies. Overall, the diagnostic performance of individual autoantibody markers was quite limited, with 86% of markers showing less than 50% sensitivity. Of note, case numbers were mostly low internal and/or external validations were rarely implemented in these studies. Therefore, one needs to be cautious when interpreting the results.

Four autoantibodies (anti-CLP peptide 104–113, anti-Mesothelin, anti-Ezrin, anti-ENOA1,2) showed reasonable diagnostic performance (sensitivity greater than 60% and specificity greater than 80%). However, one should also note that the performance of each these markers has only been reported in one study with mostly small numbers of cases and/or controls. The use of different autoantibody detection methods, different cutoff values chosen and patient samples representing different tumor stages might furthermore affect the generalizability of these findings. Therefore, validation of potential markers by independent studies is essential. Also, none of the markers have been looked at in a large-scale pancreatic cancer screening setting. Three of the four antibody recognized proteins mentioned above (Mesothelin, Ezrin, ENOA) are highly expressed in different cancers including pancreatic cancer [53–55]. Mesothelin, Ezrin and ENOA have also been linked to tumor metastasis and cancer progression. Not much has been reported on CLP (coactosin-like protein) and it is unclear if it is overexpressed or involved in pancreatic cancer. The molecular function of these proteins varies. Mesothelin is a GPI anchored cell surface protein that can promote cancer cell survival and proliferation [53], while Ezrin plays an important role in cellular processes like cell adhesion and migration and is linked to tumor metastasis [54]. Moreover high expression of Ezrin is associated with poor prognosis in different cancers including pancreatic cancer [56, 57]. ENOA (α-enolase) is a metabolic enzyme that is important for glycolysis. It is also expressed on the cell surface where it acts as a receptor for plasminogen. As seen with Ezrin and Mesothelin, ENOA has been linked to cell migration and cancer metastasis [55]. CLP can bind actin and to 5-Lipoxygenase, a key enzyme involved in the biosynthesis of the inflammatory mediators Leukotrienes [58, 59], but less is known about its biological and molecular function.

Our analysis also indicates that the combination of multiple markers (either autoantibodies in combination with tumor associated markers or multiple autoantibodies) is likely the way forward as this improves sensitivity while not dramatically affecting specificity. The challenge remains to find out which combination of markers works best and this will require additional effort to sort out. The multi-marker regression model commonly used in this context may suffer from substantial over-optimism unless appropriate internal and/or external validations are carried out, which has often not been done in the past [60]. In addition, transparent reporting of a multivariable prediction model is essential for replication of study findings by other independent researchers. Future studies following the recently proposed TRIPOD statement by Collins and colleagues would strongly improve the overall validity of research findings [61].

What remains unclear is if autoantibodies are good markers for determining early stage pancreatic cancer, which is an important issue with pancreatic cancer treatment at the moment. Two studies reported sensitivity according to tumor stage and in both studies only advanced tumor stages showed reasonable sensitivity. However, given the small sample size one has to be careful what conclusions one can draw from this and clearly more work is needed to evaluate this. Finding biomarkers that can detect early stages of pancreatic cancer is a pressing concern and not a trivial one to address. One of the included studies [26] used as a starting point a spontaneous pancreatic cancer mouse mode to identify serum autoantibodies against TAA. The benefit of the mouse model is that sample size is not a limiting factor and that one has temporal control over when to collect the samples. Using this approach, serum autoantibodies to Ezrin were shown to develop early in the pancreatic cancer murine model and also in human patients with PDAC [26]. In this case, the mouse model worked as an effective screening tool and time will tell if this approach will aid in the discovery of early stage biomarkers for pancreatic cancer.

Many of the studies used a candidate approach when choosing what autoantibodies to analyze, while others used proteomic approaches without a priori defined targets. From the list of autoantibodies examined, it is fair to say that there is no good way of predicting which markers might work. Case in point is the tumor suppressor gene p53 that has been linked to many cancers and also pancreatic cancer and therefore would be a reasonable marker to investigate. 8 studies have examined anti-p53 in pancreatic cancer, but none of them reported high enough sensitivity to support that anti-p53 on its own could serve as a good marker for detecting pancreatic cancer. So the way forward is likely large scale and unbiased screens to identify the autoantibody signatures using well-defined tumor stage samples.

Lastly, all the included studies recruited participants in a clinical setting, i.e., cases were typically clinically diagnosed patients in hospitals, and convenient controls or healthy donors were used. Various key factors regarding specimens, such as blood sample collection, storage and handling, would introduce additional bias if not well controlled [62]. The choice of cutoff values may also make the comparison between studies difficult. Additionally, there is also some variability in what data and patient characteristics were reported in the published literature. Some studies did not report important information on age or male/female ratio. While others did not provide information on what type of control samples was used. Agreement on what key factors need to be reported will help in comparing different studies and move the field forward. Also, while some studies used healthy participants as controls others used a mix of non-cancer patients, which also might make it difficult to draw comparisons between studies.

A particular challenge in diagnosing pancreatic cancer is the distinction of early pancreatic cancer from often, benign pancreatic diseases, which should receive particular attention in the selection of control groups in future studies. On the other hand, there is increasing evidence that autoantibodies against tumor-associated antigens may not be unique for specific types or locations of cancer. Possibly, autoantibodies signatures might therefore be best used as a screening tool to detect the presence of cancer in general to be followed by more specific diagnostic measures in case of a positive result.

To our knowledge, this is the first systematic literature review on serum autoantibodies as biomarkers for pancreatic cancer detection. There are some limitations that need to be considered when interpreting our review. Although we conducted a systematic search of relevant articles in two most widely-used databases and also adopted intensive cross-referencing, we cannot guarantee that all relevant studies have been identified. In addition, due to the larger heterogeneity in terms of study designs, detection methods and examined autoantibodies among included studies, a meta-analysis summarizing the diagnostic performance of markers was not meaningful.

To sum up, our review suggests that autoantibodies have the potential to be used as novel diagnostic markers for detecting pancreatic cancer possibly as part of a general cancer screening. However, current research in this area is still at a fairly early stage. More work is needed to identify promising autoantibody signatures and evaluate their diagnostic performance in detecting pancreatic cancer, especially at early stages. Given the limited diagnostic potential for single markers, multi-marker combinations are needed to enhance the overall sensitivity. Future studies adopting more rigorous study designs and reporting well-adjusted diagnostic performance characteristics in a transparent manner would contribute greatly in this research area.

## MATERIALS AND METHODS

The systematic literature review was carried out according to a predefined protocol. Reporting follows the PRISMA statement [63].

### Literature search

A systematic literature search was carried out to identify studies that evaluated serum autoantibodies produced in pancreatic cancer patients and cancer free controls. PubMed (January 1, 1950 to April 27, 2015) and ISI Web of Knowledge (January 1 1945 to April 27, 2015) were searched for relevant articles that met our inclusion and exclusion criteria. The search was done using the following keyword combinations: [(pancreatic) and (cancer or neoplasm or carcinoma or adenoma or malignancy) and (autoantibodies or antibodies) and (detection or diagnosis or biomarker) and (serum or blood or plasma)] (Supplementary Table S1). Duplicated articles were removed. The initial screen was done based upon reading of the title and abstract. Articles that were not relevant to the topic were excluded. The second round of screening involved reading of the articles in full. In addition, we also identified a number of papers from cross-referencing (Figure 1).

### Eligibility criteria

Only articles written in English were included in our review. Conference abstracts and reviews were excluded because of insufficiently reported information regarding diagnostic performance of autoantibody markers. We required that studies reported relevant information regarding diagnostic performance of autoantibody markers (e.g., sensitivity, specificity, area under the curve) for the detection of pancreatic cancer in humans as well as the numbers of cases and controls used in the studies. Studies not using cancer-free controls were further excluded.

### Data extraction

Two reviewers (KD and HC) independently read and retrieved data from the studies that met the above described inclusion and exclusion criteria. Any inconsistencies were discussed and resolved among the authors. We report the characteristics of study population (numbers of cases and controls, mean age and age range of study participants, male/female ratio and country where the study was performed), the health status of controls and the autoantibody detection method. The following diagnosis related indicators were extracted: overall and stage specific (if reported) sensitivity and specificity, area under the receiver operating characteristics curve (AUC). 95% confidence intervals (95% CI) of sensitivities and specificities were calculated using medcalc software (https://www.medcalc.net/tests/diagnostic_test.php)

## SUPPLEMENTARY MATERIALS FILE AND TABLES


