# Supplementary material for: A systematic review of serum autoantibodies as biomarkers for pancreatic cancer detection
Source: Oncotarget. 2016 Jan 31;7(10):11151–64. doi: 10.18632/oncotarget.7098 (PMC4905464; doi:10.18632/oncotarget.7098)
Supplement: Supplementary file 1 [file oncotarget-07-11151-s001.pdf]

A systematic review of serum autoantibodies as biomarkers for pancreatic cancer detection

Supplementary Materials

Supplementary File S1: The following studies were excluded for the following reasons: (**A**) *Didn't evaluate autoantibodies or diagnostic markers (n = 3):* (1) A.M. Attallah, M.M. Abdel-Aziz, A.M. El-Sayed, A.A. Tabll. Detection of serum p53 protein in patients with different gastrointestinal cancers, Cancer Detect Prev. 2003;27:127–31 [10]. (2) K.M. Kalkner, L. Ronnblom, A. Karlsson Parra, M. Bengtsson, Y. Olsson, K. Oberg. Antibodies against double-stranded DNA and development of polymyositis during treatment with interferon. QJM 1998;91:393–9 [11]. (3) S. Muro, Y. Miyake, H. Kato, K. Tsutsumi, K. Yamamoto. Serum Anti-60S Ribosomal Protein L29 Antibody as a Novel Prognostic Marker for Unresectable Pancreatic Cancer. Digestion 2015; 91:164–73 [12]. (**B**) *No cancer free controls used (n = 2):* (4) K. Angelopoulou, E.P. Diamandis. Autoantibodies against the p53 tumor suppressor gene product quantified in cancer patient serum with time-resolved immunofluorometry. Cancer J 1993 6:315–321 [13]. (5) Y. Hamanaka, Y. Suehiro, M. Fukui, K. Shikichi, K. Imai, Y. Hinoda. Circulating anti-MUC1 IgG antibodies as a favorable prognostic factor for pancreatic cancer. Int J Cancer Int J Cancer. 2003;103:97–100 [14]. (**C**) *Unable to calculate sensitivity or specificity from data provided (n = 4)*. (6) M. Kusama, K. Kusama, S. Hayashi, T. Sunouchi, L. Chu, I. Moro. Natural antibody against Thomsen-Friedenreich antigen in sera of patients with carcinomas and infectious diseases. J Nihon Univ Sch Dent. 1993;35:241–3 [15]. (7) T.H. Patwa, Y. Wang, D.M. Simeone, D.M. Lubman. Enhanced detection of autoantibodies on protein microarrays using a modified protein digestion technique. J Proteome Res. 2008;7:2553–61 [16]. (8) H. Suzuki, D. F. Graziano, J. McKolanis, O. J. Finn. Expressed Cyclin B1 Protein in Patients with Cancer and T Cell− Dependent Antibody Responses against Aberrantly Premalignant Disease. Clin Cancer Res 2005;11:1521–1526 [17]. (9) R. Talar-Wojnarowska, A. Gasiorowska, M. Olakowski, D. Dranka-Bojarowska, P. Lampe, J. Smigielski, M. Kujawiak, J. Grzegorczyk, E.Malecka-Panas. Utility of serum IgG, IgG4 and carbonic anhydrase II antibodies in distinguishing autoimmune pancreatitis from pancreatic cancer and chronic pancreatitis Advances in Medical Sciences2014 59:288–292 [18]. (**D**) *Not clear how many controls were used (n = 1)*. (10) Y. Kotera, J. D. Fontenot, G. Pecher, R. S. Metzgar, O. J. Finn. Mucin MUC-1 in Sera from Breast, Pancreatic, and Colon Cancer Humoral Immunity against a Tandem Repeat Epitope of Human Patients. Cancer Res 1994;54:2856–2860 [19].

Supplementary Table S1: Keyword search until 27th of April 2015

Search	Term	Pubmed	ISI Web of Knowledge
**1**	**Pancreatic**	**249,652**	**254,321**
2	Cancer	3,108,636	2,468,334
3	Neoplasm	2,707,896	217,198
4	Carcinoma	742,423	1,045,263
5	Adenoma	101,892	85,742
6	Malignancy	2,697,036	259,573
**7**	**2 or 3 or 4 or 5 or 6**	**3,191,728**	**3,069,111**
8	Autoantibodies	95,132	87,601
9	Antibodies	878,530	1,147,172
**10**	**8 or 9**	**884,708**	**1,180,019**
11	Detection	654,386	1,561,080
12	Diagnosis	8,797,195	1,620,078
13	Biomarker	694,776	122,117
**14**	**11 or 12 or 13**	**9,354,866**	**3,107,883**
15	Serum	908,074	1,265,874
16	Blood	3,671,969	2,188,613
17	Plasma	756,760	1,735,947
**18**	**15 or 16 or 17**	**4,249,118**	**4,489,131**
**19**	**1 and 7 and 10 and 14 and 18**	**1400**	**436**

Keyword search and number of articles found using PubMed and ISI Web of Knowledge searches.

Supplementary Table S2: Study Characteristics

First author, Year [Ref]	Country	Cases (N)/ Controls (N)	Age Range Cases/ Controls	Average Age Cases/ Controls	% Males Cases/Con-trols	Status Controls	Detection Method
Kamei, 1992 [33]	Japan	8/45	−/−	−/−	−/−	Healthy	ELISA
Marxsen, 1994 [36]	Germany	78/82	−/−	−/−	−/−	Benign pancreatic disease	ELISA
Laurent-Puig, 1995 [34]	France	29/33	−/−	−/−	−/−	Benign biliary or pancreatic disease	ELISA
Gansange, 1996 [20]	Germany	145/60	38−92/30−75	63/54	59/46	Healthy	ELISA and Western blot
Raedle, 1996 [43]	Germany	33/52	−/−	67/−	55/63	Acute and chronic pancreatitis	ELISA
Syrigos, 1996 [44]	Greece	36/21	40−82/39−77	64/63	50/57	Healthy	ELISA
Fyssas, 1997 [28]	Greece	33/40	40−82/19−76	64/62	52/63	Healthy	**
Maacke, 2002 [22]	Germany	57/86	−/−	−/−	−/−	Healthy	Westen blot
Nakatsura, 2002 [39]	Japan	8/9	−/−	−/−	−/−	Healthy	ELISA
Ohshio, 2002 [40]	Japan	82/21	−/−	−/−	61/−	Chronic pancreatitis	ELISA
Hong, 2004 [31]	USA	36/15	57−74/57−74	−/−	−/−	Healthy	Western blot
Okada, 2005 [41]	Japan	37/34	−/−	−/−	−/−	Healthy	SEREX
Xia, 2005 [49]	China	60/60	−/−	−/−	−/−	Healthy	ELISA
Muller, 2006 [37]	Germany	22/436	−/−	−/48	−/49	Healthy	Western blot
Tanaka, 2006 [46]	Japan	47/42	−/−	−/−	−/−	Healthy	Flow cytometry assay
Tanaka, 2007 [45]	Japan	40/60	−/−	65/−	78/−	Non-cancer	Flow cytometry assay
Tomaino, 2007 [47]	Italy	70/40	32−86/57−87	67/62	44/34	Healthy	2-DE gel and western blot
Johnston, 2009 [32]	USA	74/5	44−79/67−71	69/−	67/−	Healthy	ELISA

Patwa, 2009 [42]	USA	49/43	54−74/age matched	65.4/age matched	sex matched	Healthy	Protein microarray
		54/94	54−74/age matched	65.4/age matched	sex matched	Healthy	
Pekarikova, 2010 [23]	Czech Republic	55/56	59−79/23−63	72/31	58/46	Healthy	ELISA
Gnjatic, 2010 [29]	USA	60/53	31−81/38−92	63/62	68/51	Healthy	Protein microarray
Heller, 2010 [30]	Germany	34/20	−/−	65/60	76/80	Healthy	ELISA
Li, 2010 [21]`	USA	48/40	−/−	67/53	56/65	Healthy	Protein microarray
Tomaino, 2011 [48]	Italy	120/40	32−86/57−87	67/71	56/35	Healthy	ELISA
		61/63	−/−	−/−	−/−	Healthy	
		37/63	−/−	−/−	−/−	Healthy	
Bracci, 2012 [24]	USA	300/300	32−85/32−85	65/65	54/54	Not specified	Multiplex Serology
Li, 2012 [35]	China	23/23	43−83/−	65/−	65/−	Healthy	ELISA
Burfold, 2013 [25]	UK	35/247	63−70/56−66	64/61	0/0	Healthy	Glycopeptide arrays
Capello, 2013 [26]	Italy	120/40	32−86/−	67/−	56/−	Healthy	ELISA/ SERPA
		16/32	−/−	55/55	56/56	Healthy	
		69/94	42−84/−	63/−	57/−	Benign controls*	
Falco, 2013 [27]	Italy	52/44	−/−	64/59	56/70	Healthy	ELISA
Nagayoshi, 2014 [38]	Japan	37/20	43−78/−	64/−	59/−	Healthy	AlphaScreen ***
Zhu, 2015 [50]	USA	41/135	−/−	−/−	−/−	Healthy	ELISA

*Mix of healthy, chronic pancreatitis or autoimmune disease
**Semi-quantitative estimation of Bayden with passive hemagglutination
***AlphaSreeen from PerkinElmer
The table shows the key characteristics of the 31 articles included in the study: number of cases and controls, age range and average age, male/female ratio, health status of controls and autoantibody detection method.

Supplementary Table S3: Summary of studies reporting tumor stage sensitivity

First author, Year [Ref]	Antigen	Stage	No. of cases	No. of antigen positive cases	Stage specific sensitivity	*p*-value*	Overall Sensitivity	Overall Specificity
Gansange, 1996 [20]	p53	I + II	29	1	3%	$p < 0.02$	16%	100%
		III	75	20	27%	–		
		IV	41	2	5%	$p < 0.02$		
Li, 2012 [35]	p53	I	3	0	0%	–	17%	100%
		II	8	1	13%	–		
		III	5	1	20%	–		
		IV	7	2	29%	$p < 0.01$		
Li, 2012 [35]	p16	I	3	1	33%	–	30%	96%
		II	8	1	13%	–		
		III	5	2	40%	–		
		IV	7	4	57%	–		
Li, 2012 [35]	p62	I	3	0	0%	–	22%	100%
		II	8	1	13%	–		
		III	5	1	20%	–		
		IV	7	3	43%	$p < 0.01$		
Li, 2012 [35]	Survivin	I	3	0	0%	–	17%	96%
		II	8	1	13%	–		
		III	5	1	20%	–		
		IV	7	2	29%	$p < 0.01$		
Li, 2012 [35]	Koc	I	3	0	0%	–	22%	100%
		II	8	1	13%	–		
		III	5	1	20%	–		
		IV	7	3	43%	$p < 0.01$		
Li, 2012 [35]	IMP1	I	3	0	0%	–	26%	96%
		II	8	1	13%	–		
		III	5	2	40%	–		
		IV	7	3	43%	$p < 0.01$		
Li, 2012 [35]	Panel of 6 antibody markers**	I	3	1	33%	–	61%	87%
		II	8	3	38%	–		
		III	5	4	80%	–		
		IV	7	6	86%	$p < 0.01$		

Abbreviations: IMP1: IGF-II mRNA binding protein; Koc: KH-domain containing protein over expressed in cancer
**p*-value represents the difference of positivity rate between cases and controls
**p53+p16+p62+Survivin+Koc+IMP1
Two studies provided diagnostic performance of autoantibodies in defined clinical tumor stages.
